# The IUPHAR/BPS Guide to PHARMACOLOGY in 2018: updates and expansion to encompass the new guide to IMMUNOPHARMACOLOGY

**DOI:** 10.1093/nar/gkx1121

**Published:** 2017-11-15

**Authors:** Simon D Harding, Joanna L Sharman, Elena Faccenda, Chris Southan, Adam J Pawson, Sam Ireland, Alasdair J G Gray, Liam Bruce, Stephen P H Alexander, Stephen Anderton, Clare Bryant, Anthony P Davenport, Christian Doerig, Doriano Fabbro, Francesca Levi-Schaffer, Michael Spedding, Jamie A Davies, NC-IUPHAR

**Affiliations:** Deanery of Biomedical Sciences, University of Edinburgh, Edinburgh EH8 9XD, UK; Department of Structural & Molecular Biology, University College London, London WC1E 6BT, UK; School of Mathematical and Computer Sciences, Heriot-Watt University, Edinburgh EH14 4AS, UK; School of Life Sciences, University of Nottingham Medical School, Nottingham NG7 2UH, UK; MRC Centre for inflammation Research, University of Edinburgh, Edinburgh EH16 4TJ, UK; Department of Veterinary Medicine, University of Cambridge, Cambridge CB3 0ES, UK; Experimental Medicine and Immunotherapeutics, University of Cambridge, Cambridge CB2 0QQ, UK; Department of Microbiology, Monash University, Clayton 3800, Australia; PIQUR Therapeutics, Basel 4057, Switzerland; Pharmacology and Experimental Therapeutics Unit, School of Pharmacy, Institute for Drug Research, Hebrew University of Jerusalem, Jerusalem 9112102, Israel; Spedding Research Solutions SAS, Le Vésinet 78110, France

## Abstract

The IUPHAR/BPS Guide to PHARMACOLOGY (GtoPdb, www.guidetopharmacology.org) and its precursor IUPHAR-DB, have captured expert-curated interactions between targets and ligands from selected papers in pharmacology and drug discovery since 2003. This resource continues to be developed in conjunction with the International Union of Basic and Clinical Pharmacology (IUPHAR) and the British Pharmacological Society (BPS). As previously described, our unique model of content selection and quality control is based on 96 target-class subcommittees comprising 512 scientists collaborating with in-house curators. This update describes content expansion, new features and interoperability improvements introduced in the 10 releases since August 2015. Our relationship matrix now describes ∼9000 ligands, ∼15 000 binding constants, ∼6000 papers and ∼1700 human proteins. As an important addition, we also introduce our newly funded project for the Guide to IMMUNOPHARMACOLOGY (GtoImmuPdb, www.guidetoimmunopharmacology.org). This has been ‘forked’ from the well-established GtoPdb data model and expanded into new types of data related to the immune system and inflammatory processes. This includes new ligands, targets, pathways, cell types and diseases for which we are recruiting new IUPHAR expert committees. Designed as an immunopharmacological gateway, it also has an emphasis on potential therapeutic interventions.

## INTRODUCTION

The International Union of Basic and Clinical Pharmacology/British Pharmacological Society IUPHAR/BPS Guide to PHARMACOLOGY (GtoPdb, www.guidetopharmacology.org) is an expert-curated resource of ligand–activity–target relationships, selected from high-quality pharmacological and medicinal chemistry literature. It has its origins in IUPHAR-DB, first compiled in 2003 and focused on receptors and channels (1–3). From 2012 to 2015, the scope expanded to define the data-supported druggable genome and the resource was re-named GtoPdb (4,5). This phase added many new target families and consolidated ligand-to-target relationships for approved drugs and clinical candidates. Over the last 2 years, under the guidance of the IUPHAR Committee on Receptor Nomenclature and Drug Classification (NC-IUPHAR) and its 96 expanded subcommittees (comprising 512 international scientists), we have added quantitative ligand–target relationships showing promise for future drug discovery. We have also enhanced interoperability with other resources and added features to help users access structure-activity relationship (SAR) data and to explore species differences using a new ligand activity visualization tool.

From 2015, we have addressed the priority area of immunity, inflammation and infection (6–9). Most chronic diseases, including ageing, have an immune-inflammatory component (10); auto-immunity is a serious problem (11,12), and the progress of infections depends on immune and inflammatory responses (13). There is also increased interest in anti-tumour immune activity, the importance of which is underlined by a new partnership between IUPHAR and the International Union of Immunological Sciences (IUIS) to create standard tools and nomenclature. Critical to facilitate this is a strong connection between the immunity, inflammation and infection research and the pharmacological communities, with easy bidirectional data flow. In particular, pharmacological information is difficult for immunologists to access without chemoinformatic expertise not typically present in immunology labs. Immunology is supported by excellent databases—e.g. ImmPort (www.immport.org), ImmGen (www.immgen.org), InnateDB (www.innatedb.ca/), Reactome (www.reactome.org) and IMGT (www.imgt.org)—but these do not provide easy links to pharmacological data. The development of the ‘IUPHAR Guide to IMMUNOPHARMACOLOGY’ (or GtoImmuPdb for short), with an immunologist-friendly portal, aims to address this gap in information exchange. All data added for the immunopharmacology project are also available from the original GtoPdb site and *vice versa*.

## GUIDE TO PHARMACOLOGY CURATION AND EXPANSION

The value of the database arises from our approach to document selection, data curation and annotation. Our curation ‘rules’ are better seen as flexible guidelines because expert judgement is exercised at all stages of the process, with the overriding pragmatic principle being to expand content value while maintaining stringency. This quality-centric, ‘small data’ approach contrasts with automated data-mining, large-scale curation operations and ‘big data’ amalgamation efforts. Curation includes checking and fixing author errors (e.g. μM versus nM), adding PubChem cross-pointers between unresolved stereoisomers to the appropriate stable R and S forms, and splitting salts and parents for cheminformatic consistency while also cross-pointing to reported salt forms (e.g. specified by the United States Adopted Names (USAN) or Food and Drug Administration (FDA) drug information sheet). We have recently simplified the representation of activity ranges to one-value-one-reference relationships, extending this principle for patent-only entries and adapting legacy data to this form.

### Content

#### Targets

A ‘target’ record in our database resolves to a UniProtKB/SwissProt Accession as its primary identifier. Since our 2016 publication (5), target expansion has been mainly for GtoImmuPdb. Specifically-added immunity, inflammation and infection targets include the following the pattern recognition receptors/proteins of the innate immune response:

RIG-I-like receptor family (www.guidetopharmacology.org/GRAC/FamilyDisplayForward?familyId=940)
Absent in melanoma-like receptors (www.guidetopharmacology.org/GRAC/FamilyDisplayForward?familyId=942)
C-type lectin-like receptors (www.guidetopharmacology.org/GRAC/FamilyDisplayForward?familyId=945)
Other pattern recognition receptors (www.guidetoimmunopharmacology.org/GRAC/FamilyDisplayForward?familyId=929)

Immune checkpoint protein targets have been expanded and aggregated in new families, for example Immune checkpoint catalytic receptors (www.guidetopharmacology.org/GRAC/FamilyDisplayForward?familyId=953) and ‘Other immune checkpoint proteins’ (www.guidetopharmacology.org/GRAC/FamilyDisplayForward?familyId=949). This allows us to capture the action of these proteins as both immune system regulators and as drug targets. Another new target family is Butyrophilin and butyrophilin-like proteins were added for their increasingly indicated roles in gamma-delta T cells (14). As part of our ongoing updates of the GtoPdb, we have added other, non-immune targets, including the aryl hydrocarbon receptor and Piezo channels.

**Figure 1.**
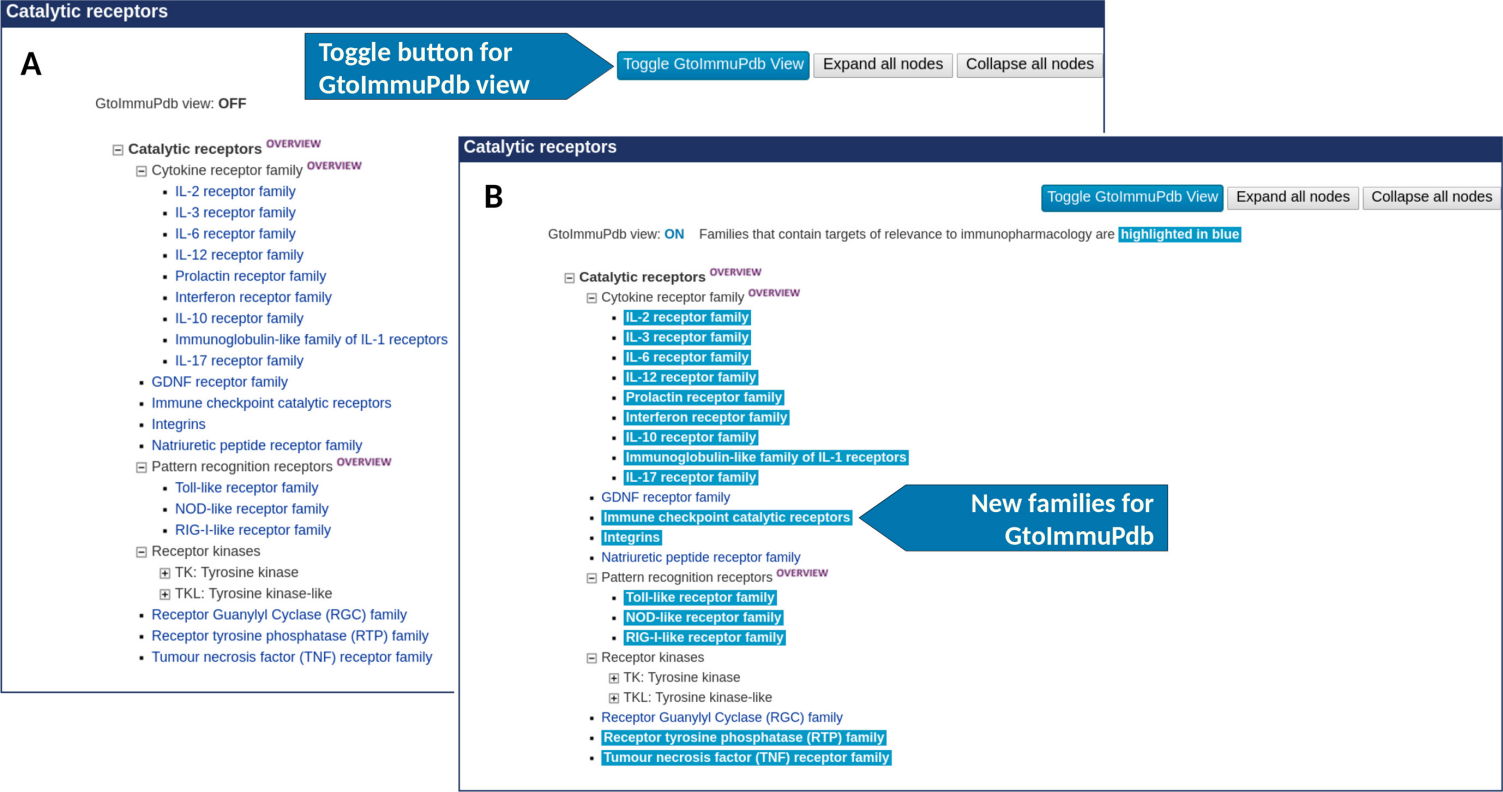
The hierarchical listing for the catalytic receptor families and subfamilies. (**A**) Shows the GtoPdb view and (**B**) shows it with the Guide to IMMUNOPHARMACOLOGY (GtoImmuPdb) view switched on, which highlights families containing targets of immunological relevance. A toggle button enables switching between GtoPdb and GtoImmuPdb views, the only difference being that the GtoPdb view does not have highlighting.

Our targets are organized into several classes (Table 1A), accessible through the website, each divided into families and subfamilies (Figure 1). Classifications can overlap, for example ‘Immune checkpoint proteins’ are in both the ‘Catalytic receptor’ and the ‘Other protein target’ classes. Joining these different target types is useful to immunologists, given the proteins’ shared roles in regulating T cells and macrophages.

**Table 1.** Guide to PHARMACOLOGY database counts for targets, ligands and interactions from database release 2017.5

	GtoPdb 2016	GtoPdb 2018 (±2016)	GtoImmuPdb 2018
**A. Target class content. Human UniProtKB accession counts**			
7TM receptors*	395	395 (0)	86
Nuclear hormone receptors	48	48 (0)	6
Catalytic receptors	239	243 (+4)	107
Ligand-gated ion channels	84	81 (-3)	3
Voltage-gated ion channels	141	144 (+3)	17
Other ion channels	47	49 (+2)	0
Transporters	508	509 (+1)	5
Enzymes (all)	1164	1184 (+20)	134
*Kinases*	539	546 (+7)	79
*Proteases*	240	243 (+3)	32
Other proteins	135	174 (+39)	64
Total number of targets	2761	2834 (+73)	420**

**B. Ligand category counts**			
Synthetic organics	5055	5807 (+752)	449
Metabolites	582	584 (+2)	23
Endogenous peptides	759	782 (+23)	176
Other peptides including synthetic peptides	1222	1297 (+75)	37
Natural products	234	247 (+13)	9
Antibodies	138	223 (+85)	121
Inorganics	34	38 (+4)	1
Approved drugs	1233	1334 (+101)	208
Withdrawn drugs	67	67 (0)	11
Ligands with INNs	1882	2114 (+232)	336
PubChem CIDs	6037	6702 (+665)	484
PubChem SIDs	8024	8978 (+954)	816
Total number of ligands	8024	8978 (+954)	816

**C. Interaction counts**			
Human targets with ligand interactions	1505	1684 (+179)	390
Human targets with quantitative ligand interactions	1228	1431 (+203)	321
Human targets with approved drug interactions	554	563 (+9)	152
Primary targets*** with approved drug interactions	312	313 (+1)	91
Ligands with target interactions	6796	7663 (+867)	718
Ligands with quantitative interactions (*approved drugs*)	5860	6716 (+856)	553
	*738*	*824 (+86)*	*138*
Ligands with clinical use summaries (approved drugs)	1724	2089 (+365)	423
	*1231*	*1332 (+101)*	*208*
Number of binding constants	44 691	46 488 (+1797)	23 304
Number of binding constants curated from the literature	13 484	15 281 (+1797)	10 964
References	27 880	31 733 (+3933)	

* Not all the 7TM receptor records are unequivocally assigned as GPCRs, but for convenience we refer to these generally as GPCRs in the text.

** Thirty-five targets are tagged in GtoImmuPdb but have no Human UniProtKB accession. Thirty-four of these are complexes (only subunits have UniProtKB accessions, and 1 that only has a mouse accession).

*** Primary target indicates the dominant Molecular Mechanism of Action (MMOA)

**** An interaction is only considered part of GtoImmuPdb where both the target and ligand are tagged as relevant to immunopharmacology. The table shows just under one quarter of all the curated interactions in GtoPdb involve targets and ligands of immunological relevance (we are still in the process of identifying these so it is likely to increase).

The table includes a comparison to the figures from the 2016 update (5), and a breakdown for the GtoImmuPdb dataset. Categories are not mutually exclusive and targets and ligands can fall into more than one, therefore totals are not the sum of all other rows.

#### Ligands

Criteria for including a ligand remain focused on well-characterized, quantitative interactions with protein targets (Table 1B). Of the 816 ligands tagged as having relevance to immunopharmacology (see GtoImmuPdb Expansion-‘Content and curation’ section for details), ∼30% have been added since the last update. As previously, ligands without an experimentally-verified molecular mechanism of action, but where potential efficacy is clearly described in papers, are also captured. There are new ligand groups, for example Immune checkpoint modulators (www.guidetopharmacology.org/GRAC/FamilyDisplayForward?familyId=969), which group disparate ligands with common functionality. We have extended the database schema to group some ligands into families using the information on Hugo Gene Nomenclature Committee (HGNC) gene family pages (www.genenames.org/cgi-bin/genefamilies/) (15,16); examples include cytokines and chemokines. A new family listing page exists under the ‘Ligands’ sub menu of the main navigation bar and also from the ligand list and individual ligand pages (www.guidetopharmacology.org/GRAC/LigandFamiliesForward).

#### Interactions and database growth

**Figure 2.**
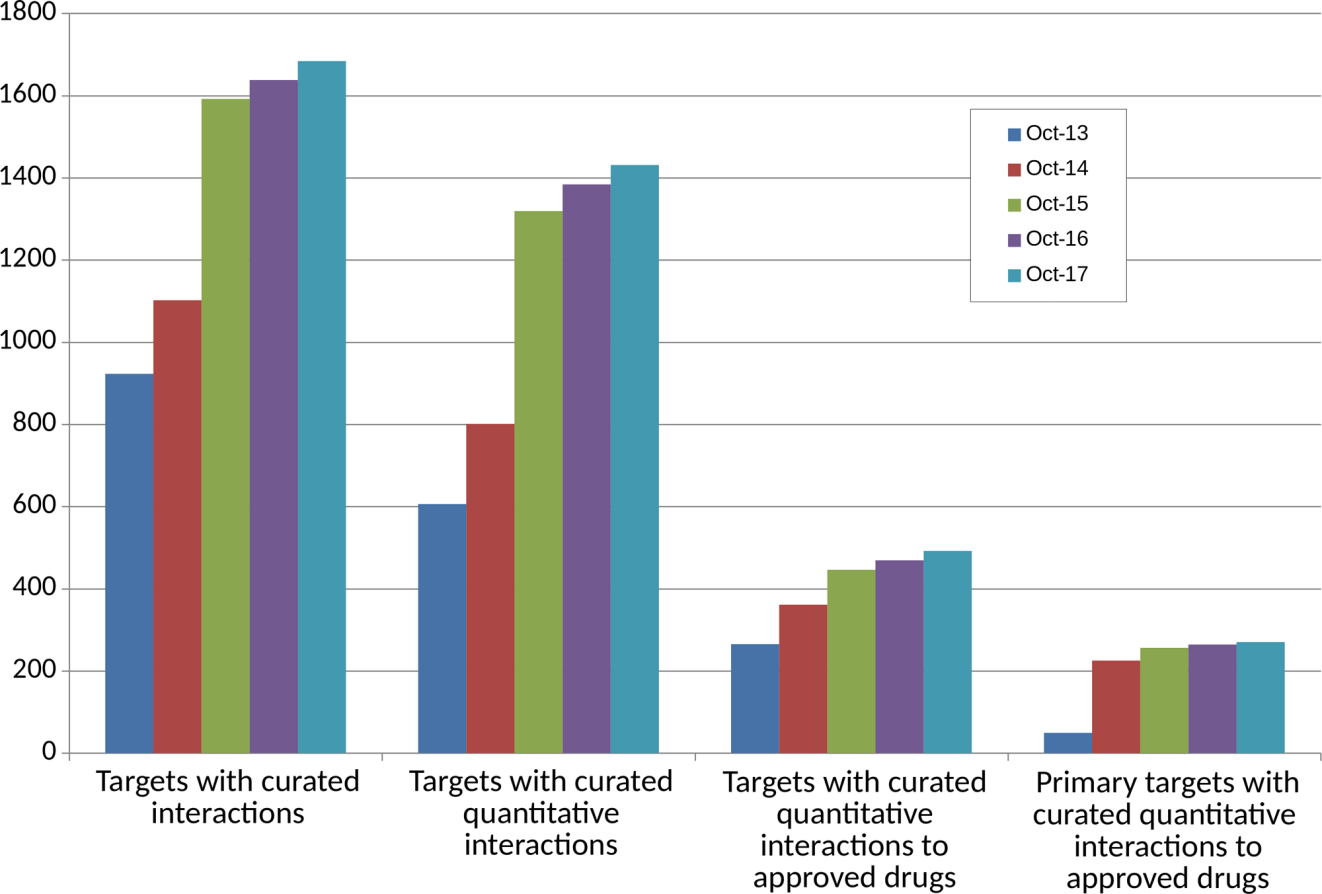
Human interaction data growth since 2013. The first (left-most) chart shows the number of human targets with curated ligand interactions while the second chart includes only those targets that are supported by quantitative data. The third chart shows the number of target data-supported interactions to approved drugs and the fourth chart shows primary targets of those drugs.

Table 1C and Figure 2 show the expansion of curated interaction data across targets and ligands. Most targets added over the past two years fall into the ‘Enzyme’ and ‘Other protein’ target categories.

#### PubChem content

Our PubChem integration strategy has been previously outlined (5). Since 2015 we have made nine PubChem submissions for new releases of our database. For 2017.5 (see release notes https://blog.guidetopharmacology.org/2017/08/22/database-release-2017-5/) we now have 8978 Substance Identifiers (SIDs) (PubChem query ‘IUPHAR/BPS Guide to PHARMACOLOGY’[SourceName]). We submit within days of our public release but it can take PubChem a few days to process a new submission and several weeks to complete the more computationally intensive relationship mappings (e.g. 3D neighbours).

**Figure 3.**
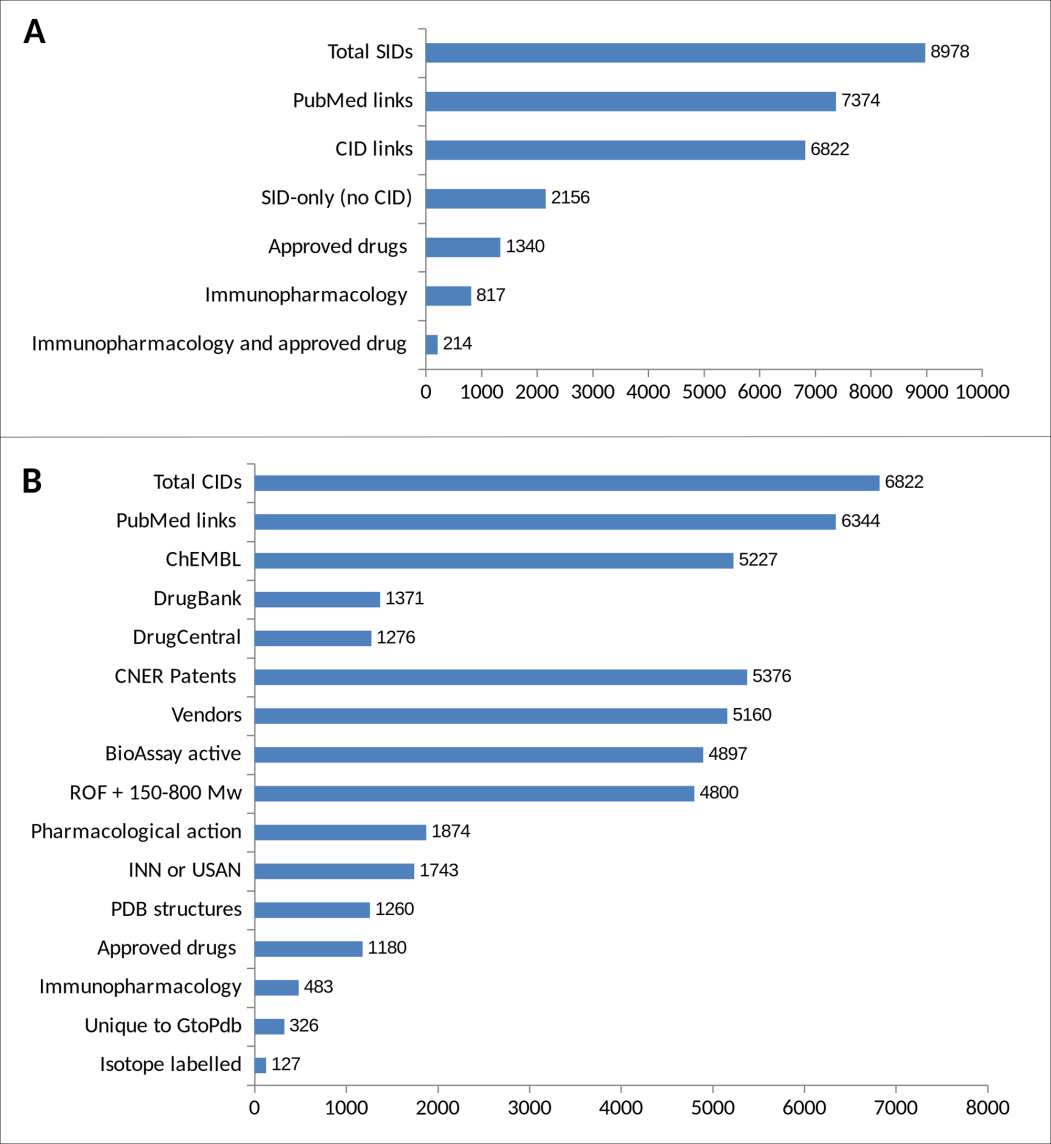
Category breakdown at the SID (**A**) and CID (**B**) level for GtoPdb PubChem entries. Note, in panel A, that the intersect between approved drug and immunopharmacology is derived from our curation of publications suggesting the association, but are not necessarily approved for immunological clinical indications.

Figure 3 shows an analysis of our content in PubChem. For the SIDs (Figure 3A), we have extended our SID comments to be able to identify ‘approved drug’ and ‘immunopharmacology’ subsets. Submission to PubChem allows users the useful ability to compare different sources, using filters for ‘slicing and dicing’ (17). Doing so highlights our complementarity to other sources, such as GtoPdb having 1595 CID structures that ChEMBL does not (Figure 3B). The same figure shows that in the case of both DrugBank (18) and DrugCentral (19) there are ∼5500 CIDs unique to just GtoPdb. A more detailed discussion of GtoPdb PubChem content and comparisons to other chemical sources can be found in our blog (https://blog.guidetopharmacology.org/2017/10/18/gtopdb-nar-database-issue-2018-pubchem-content/).

### Website enhancements

#### BLAST

We have extended the GtoPdb query tools with BLAST (20) sequence-based searching of targets (www.guidetopharmacology.org/blast/), bypassing ambiguities of protein names. Users can BLAST a query sequence against GtoPdb targets either using a polypeptide or a nucleotide sequence, which will be automatically translated in all possible frames before querying. There is the option of supplying either FASTA sequences or plain-text sequences, pasted directly into the search box or uploaded. There are various controls to filter by species, number of hits and maximum Expect value (E).

#### SAR data

To facilitate SAR studies with ligand sets for individual targets we have added the ability to download SAR data in comma-separated values (CSV) format. A link to an SAR file has been added to every target page above the tables of ligand binding data. To access SAR data for all targets, we point users to our download page (www.guidetopharmacology.org/download.jsp) where they can download a CSV file containing all interactions.

#### Visualising ligand activity across species

**Figure 4.**
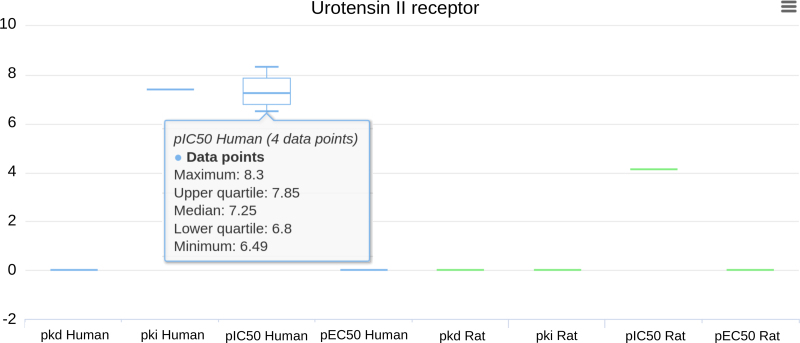
Ligand activity chart for palosuran (ACT-058362, GtoPdb ligand ID 3516), a small molecule urotensin II receptor antagonist. Hovering the mouse over the chart on a datapoint gives the median and range if more than one value is reported for the parameters shown on the horizontal axis. Zero indicates no data are available for that parameter. In the example, the results show the IC50 value for palosuran inhibition of binding was about ∼5000-fold lower in rats compared with humans, showing a major difference between the two species.

Many pharmacological parameters are reported in results sections of papers and cannot easily be found by searching of PubMed (https://www.ncbi.nlm.nih.gov/pubmed/) abstracts. Curated databases such as GtoPdb and ChEMBL (21) extract these parameters into a database format which facilitates findability and re-use. We have introduced interactive charts to summarize ligand activity at targets and compare pharmacological parameters across species. The bioactivity visualization tool uses data from GtoPdb and ChEMBL to present box plots summarising reported activities for a ligand at different targets. The charts show various pharmacological parameters expressed as -log_10_. For functional assays, potency values may include pIC_50_ or pEC_50_ values of agonists; pA_2_ or pK_B_ values for antagonists. For ligand binding assays, values may be reported as pK_d_, pK_i_ or pIC_50_. Pharmacological parameters are defined in NC-IUPHAR’s terms and symbols publication (22). The plots for each species are given a unique colour, which allows species differences to be quickly visualized (Figure 4).

Further information on assay details, references, PubMed links and source databases are in tables below the graphs. GtoPdb ligand binding data are supplemented with additional values from ChEMBL (currently version 23, updated when a new ChEMBL version is released) because, while GtoPdb primarily focuses on human targets with some supporting rodent data, ChEMBL covers a wider range of species reported in medicinal chemistry literature. Before including data from ChEMBL we applied filters, ensuring only single protein targets or protein complexes were included, normalising disparate pharmacological parameters to one of the five activity types above, converting raw data to -log_10_ values, and ignoring assays not marked as binding (B) or functional (F), as well as large scale screening data and anything with the ‘outside typical range’ warning flag.

#### NC-IUPHAR and GtoPdb faculty pages

NC-IUPHAR subcommittees reviewing GtoPdb data have, over 14 years, recorded contributions from over 800 scientists. To recognize their invaluable contributions, we have added contributor faculty pages, with individual profiles listing details of subcommittee membership and database contributions, institutional address, ORCIDs and external profile links such as departmental home pages, reference lists and social network profiles. The information is all entirely optional and provided by the individual, so some profiles are more complete than others. Contributor profiles may be accessed by clicking on a contributor’s name in citations, on the contributor list (www.guidetopharmacology.org/GRAC/ContributorListForward) or via a database search. The new faculty pages also provide users with greater insight into the data review process and roles of subcommittees.

## GUIDE TO IMMUNOPHARMACOLOGY

The Guide to IMMUNOPHARMACOLOGY (GtoImmuPdb) expansion has extended the database to incorporate data types relevant to immunopharmacology, and associate these with existing target and ligand entities. This enrichment includes data on immunological processes or pathways, cell types of relevance to immunology, and diseases related to the immune/inflammatory system. We have already described the new targets and ligands included as part of GtoImmuPdb. Here we describe methodology of tagging new and existing targets and ligands along with the major data type expansions.

### Content and curation

The immunopharmacology extension necessitated adaptation of the selection criteria. We have been supported in identifying key papers by new NC-IUPHAR committees, and we expect to gather further feedback from users on the papers they would like to include and/or remove.

Twitter is useful both for following a range of immunology journals and for broader alerting services such ImmuneRegulationNews (https://twitter.com/Immune_News), Human Immunology News (www.humanimmunologynews.com) and the British Society for Immunology (https://twitter.com/britsocimm). We have introduced an open pre-curation and contextual document triage using ‘social tagging’ via CiteUlike (23). The current list of over 700 immunopharmacology papers can be browsed via http://www.citeulike.org/tag/immpharm. These have an approximately equal split between context papers (target reviews and mechanistic immunology, e.g. (http://www.citeulike.org/user/efaccenda/article/14420478)) and those specifically designated for subsequent curatorial extraction. The former are collated under an immunopharmacology further reading page (http://www.guidetoimmunopharmacology.org/GRAC/ImmunoFurtherReadingForward). For the latter, pre-curation notes are added in the comments section (e.g. http://www.citeulike.org/user/cdsouthan/article/14414220), with links to the database added post-curation. The ‘curation queue’ can be viewed at (http://www.citeulike.org/user/cdsouthan/tag/tobecurated).

In setting up GtoImmuPdb we had many detailed curatorial aspects to consider but four are especially important. The first is that there are no unique ligand or target entities since these are all subsumed into GtoPdb (and counted in our statistics sections). This is a pragmatic consequence of immunopharmacology being a subset of pharmacology. The second is that we have made greater use of both the Gene Ontology (GO) (24,25) (www.geneontology.org) and the Cell Ontology (CO) (26) (http://obofoundry/ontology/cl.html) for classifying targets. The third is we have introduced new levels of disease linking, including associating ligands directly to diseases for the first time. The fourth is that since some existing targets are multi-purpose, we needed to maintain a clear distinction between potential GtoImmuPdb targets (e.g. identified based on GO annotations and/or speculative comments in reviews) and those where the papers we curated have made an explicit experimental link in the form of ligands tested in systems relevant to the declared goal of advancing to a therapeutic application in immunology or inflammation. The necessity for this division is particularly important for kinases in immune system cells that can be targeted for cancer (e.g. antiproliferative) as well as immune system modulation.

### Identifying targets and ligands of relevance to immunopharmacology in GtoPdb

The initial step was to identify within GtoPdb all targets and ligands relevant to immunopharmacology. We introduced a new GtoImmuPdb tag for targets and ligands, and added free-text comments describing reasons for inclusion and information about the role the target or ligand plays in immunopharmacology. Although the initial process used an automatic pipeline (see below) to identify relevant entities, all the data were reviewed by curators (supported by the appropriate committees or collaborators) before being included in GtoImmuPdb.

The review process focused on three main rationales:

- Known immune related targets and ligands (e.g. histamine receptors/antihistamine drugs, glucocorticoid receptor/corticosteroid drugs, protein kinases and their inhibitors used for immunology/inflammatory indications, targets of approved monoclonals and other biologics for immunology/inflammatory/immune-oncology indications).
- Advice from our collaborators, who suggested several targets on which to focus as proof-of-concept (e.g. Bruton tyrosine kinase/Ibrutinib and other inhibitors, colony stimulating factor 1 receptor and associated pharmacological agents, Janus kinases and associated pharmacological agents).
- Using GO annotations and literature reviews to prioritize immunological targets for curation (see below).

The counts of GtoImmuPdb tagged targets and ligands are shown in Table 1, with the full list provided on the website (http://www.guidetoimmunopharmacology.org/immuno/immunoHelpPage.jsp#downloads).

Curators continually review literature covering immunology and inflammation using the triage methodology described above. New targets and ligands are added to the database as new evidence emerges. We also review clinical developments in immunity/inflammation/immuno-oncology fields. This includes evaluating clinical development pipelines, identifying lead compounds, their molecular targets and pharmacological data (which can involve interrogation of patent literature where peer reviewed disclosure is not yet available).

### Incorporating immunological process data

The first step was to determine, in consultation with immunologists, the major processes relevant to immunology. These top-level categories form the basis of organising, navigating and searching for immunological process data (Table 2). Existing targets in GtoPdb were then organized under the relevant process categories.

**Table 2.** GtoImmuPdb process category with count of human UniProtKB proteins assigned to them based on GO annotations

Process category	GtoPdb human UniProtKB	Target-GO annotations
Barrier integrity	40	52
Inflammation	576	1277
Antigen presentation	158	226
T cell (activation)	172	345
B cell (activation)	136	222
Immune regulation	435	1072
Tissue repair	18	18
Immune system development	199	350
Cytokine production and signalling	390	979
Chemotaxis and migration	229	382
Cellular signalling	448	1079

**Figure 5.**
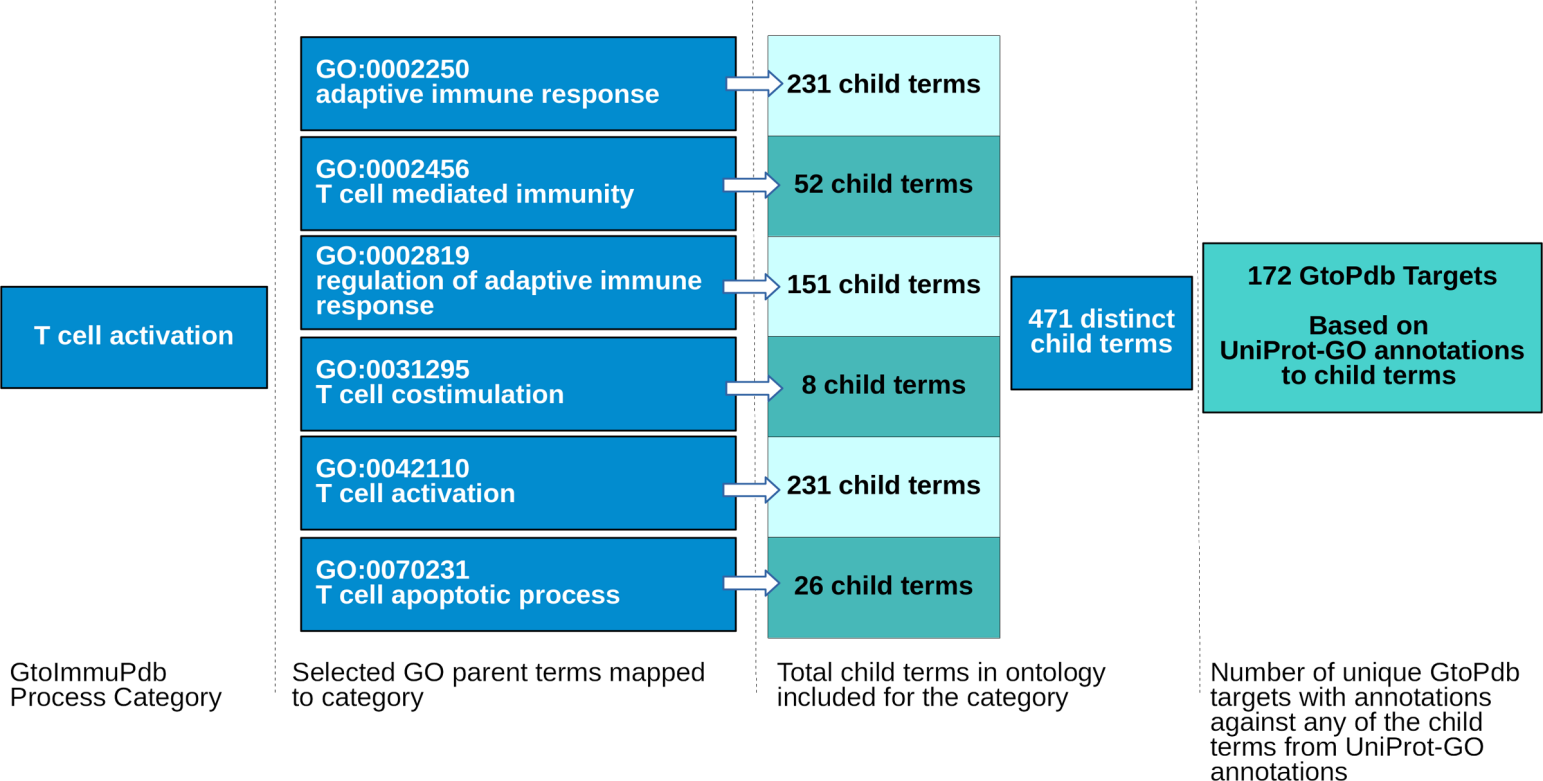
Illustrating the mapping of GO parent terms to the GtoImmuPdb top-level process category, T-cell activation. Six parent terms are mapped, which encompasses 471 distinct child terms. Using GO annotations to UniProt we find that there are 172 human targets in GtoPdb with annotations to one or more of those 471 GO terms (see Table 2).

The top-level process categories are underpinned by associating sets of GO immune and inflammatory biological process terms (27) to each category. This serves two functions; it gives a controlled vocabulary and external identifiers which support data interoperability, and enabled an initial auto-curation of targets to the top-level process categories by using their GO annotations to classify them. Relevant parent GO terms, and all their child terms, were mapped to each category. This is illustrated in Figure 5 which shows the parent GO terms (and counts of child terms) mapped to the T cell activation process category.

GO terms and IDs related to immune and inflammatory processes were obtained from the GO Consortium (http://purl.obolibrary.org/obo/go.obo) and used to populate database tables. We captured all *is_a* relationships between the terms to support inferred searching by GO terms. This means a search on a parent term will also include any of its children.

Target-GO annotations were downloaded from UniProtKB (2017_08 release) (28) and parsed into the database, along with GO evidence codes (www.geneontology.org/page/guide-go-evidence-codes). GO evidence code indicate the level of support for annotations to terms, which allows users and curators to judge the strength of the evidence for the association. Associations where the evidence is tenuous, such as for the IEA (Inferred by Electronic Analysis) code, can be split (this is the only evidence code that is assigned automatically by GO, without curatorial judgement).

The set of target-GO annotations was used to automatically assign targets to the top-level categories. Our curators then reviewed the annotations. Table 2 summarizes the total number of GO annotations to targets, and the number of UniProtKB proteins, under each category. These mappings can be downloaded from the website (http://www.guidetoimmunopharmacology.org/immuno/immunoHelpPage.jsp#downloads). All process associations can be browsed from the Immuno Process association pages (www.guidetoimmunopharmacology.org/GRAC/ProcessesForward). In order to keep GO annotations up-to-date with the latest UniProtKB release, we have semi-automated scripts that are run upon each database release.

### Incorporating immune system cell type data

We used a similar approach to incorporate cell type data. We sought input from immunologists to establish a set of top-level cell type categories, against which targets in GtoImmuPdb can be annotated. For example mast cells are included due to their relevance in anti-allergic therapies (29), and there is a category for innate lymphoid cells, reflecting the growing understanding of their role within the innate immune system in the control of tissue homeostasis, infection, inflammation, metabolic disease and cancer (30). We have under-pinned these categories with terms from the CO. The full list of cell type categories, their CO term assignments, and a breakdown of targets in each category are given in Table 3.

**Table 3.** GtoImmuPdb cell type top-level categories, with associated Cell Ontology terms and count of distinct targets annotated to each category

Cell type category	Cell Ontology terms	Targets annotated
B cells	CL:0000945 lymphocyte of B lineage	32
T cells	CL:0000789 alpha-beta T cell	39
	CL:0000815 regulatory T cell	
	CL:0000911 effector T cell	
Dendritic cells	CL:0000451 dendritic cell	29
Other T cells	CL:0000798 gamma-delta T cell	1
	CL:0000814 mature NK T cell	
	CL:0000898 naive T cell	
	CL:0000940 mucosal invariant T cell	
Macrophages and monocytes	CL:0000235 macrophage	37
	CL:0000576 monocyte	37
Granulocytes	CL:0000094 granulocyte	34
Natural killer cells	CL:0000623 natural killer cell	21
Mast cells	CL:0000097 mast cell	26
Innate lymphoid cells	CL:0001065 innate lymphoid cell	0
Stromal cells	CL:0000499 stromal cell	0

T cells are split into two categories, ‘T cells’ and ‘other T cells’, which was to keep the regulatory T cells together and distinct from other T cell types.

The top-level categories broadly correlate with the CO terminology. We then selected relevant parent terms and mapped these to our top-level categories. This enables inferred searches to be run via any CO term, finding targets associated to that term or its children. It also supports higher resolution annotation and gives a controlled vocabulary and external identifiers. Cell type associations can be browsed from the Immuno Cell Type association pages (www.guidetoimmunopharmacology.org/GRAC/CelltypesForward).

### Associating diseases on immunological relevance

GtoPdb already contained data on diseases, including clinically-relevant mutations and pathophysiological roles of targets. We have extended the schema to include specific associations of targets and ligands with diseases of immunological relevance. Curatorial comments describe the link between the target or ligand and the disease, along with supporting literature references. New disease summary pages link information on approved drugs, clinical use and primary targets to disease associations, bringing new perspectives to immunopharmacology data.

We consulted several resources—OMIM (31) (www.omim.org/), Orphanet (www.orpha.net) and the Disease Ontology (32) (http://disease-ontology.org/). Incorporating their disease terminology provides a controlled vocabulary, and cross-references our disease associations to other resources.

The GtoPdb database contains over 2000 disease terms (including synonyms). We have so far matched over 300 to ligands of immunological relevance. Curation of targets against immunological disease is in its infancy, but already contains associations to 17 immunological diseases. As expected, these include those under particularly active investigation (as judged by the current literature output), such as asthma, rheumatoid arthritis, inflammatory bowel disease, chronic obstructive pulmonary disease, sepsis and fibrosis. A full breakdown of the disease associations can be viewed at www.guidetoimmunopharmacology.org/GRAC/ImmunoDiseaseListForward?type=target.

## WEBSITE

The GtoPdb website has been extended to surface new data types and incorporate them into existing search and browse mechanisms. We have not developed a new resource, but have extended the existing database and website with a new ‘layer’ of immunological data and an interface for immunologist users, the ‘GtoImmuPdb view’ of the data. This view highlights content of immunological relevance and prioritizes immunological data in search results and displays. In addition, we created a new entry portal (at www.guidetoimmunopharmacology.org) which provides access points to immunological data.

At this stage, the Guide to IMMUNOPHARMACOLOGY is in beta-release (v2.0): although it contains most features and functionality expected in the full public release (due in 2018), it remains under development and may contain minor bugs and/or portions not yet optimized. Full details on navigating the new features of GtoImmuPdb are in our website tutorial (www.guidetoimmunopharmacology.org/immuno/docs/GtoImmuPdb_Tutorial.pdf).

### Portal

The GtoImmuPdb portal is intended to provide a new access point to the database, with quick access to the data types of most relevance to immunopharmacology. The portal is styled after its parent GtoPdb, but with its own distinctive logo, header and colour scheme. This ensures that it is intuitively familiar to GtoPdb users while at the same time maintaining its own identity. We have also used the new colour scheme to highlight content of relevance to immunopharmacology within the existing GtoPdb interface. In several places toggle buttons allow users to switch between the new GtoImmuPdb view and the existing GtoPdb view (see Figure 1). The portal provides easy access to the new data types via panels on the portal home page. Detailed descriptions of navigating and viewing data in GtoImmuPdb are given in the next few sections. A revised menu bar has been developed for GtoImmuPdb that includes links and resources specific for GtoImmuPdb, including links to the new Process, Cell Type and Disease data. The site-wide search box in the top-right has also been modified to search across the new data types (see the ‘Searching GtoImmuPdb’ section for details).

### Process associations to targets

Clicking one of the top-level categories on the Processes panel displays a new page listing all targets associated with that process (www.guidetoimmunopharmacology.org/GRAC/ObjectImmProcessListForward?immprocess=2). For each target, curators’ comments give detailed information about the target’s relevance to the immunological process and immunopharmacology in general. Immune-relevant GO annotations to the targets, including the GO ID and evidence code, are displayed, enabling users to assess the strength of the association. Targets are organized into sections, one for each main target class; quick links at the top of the page allow users to jump to each of these sections. The page also has a pull-down menu containing all the top-level process categories, enabling the user to easily switch between different categories. The process associations to target pages can also be accessed via the main menu bar and there is a processes home page (www.guidetoimmunopharmacology.org/GRAC/ProcessesForward) that summarizes each of the categories, including lists of the GO parent terms that are used to define them.

### Cell type associations to targets

**Figure 6.**
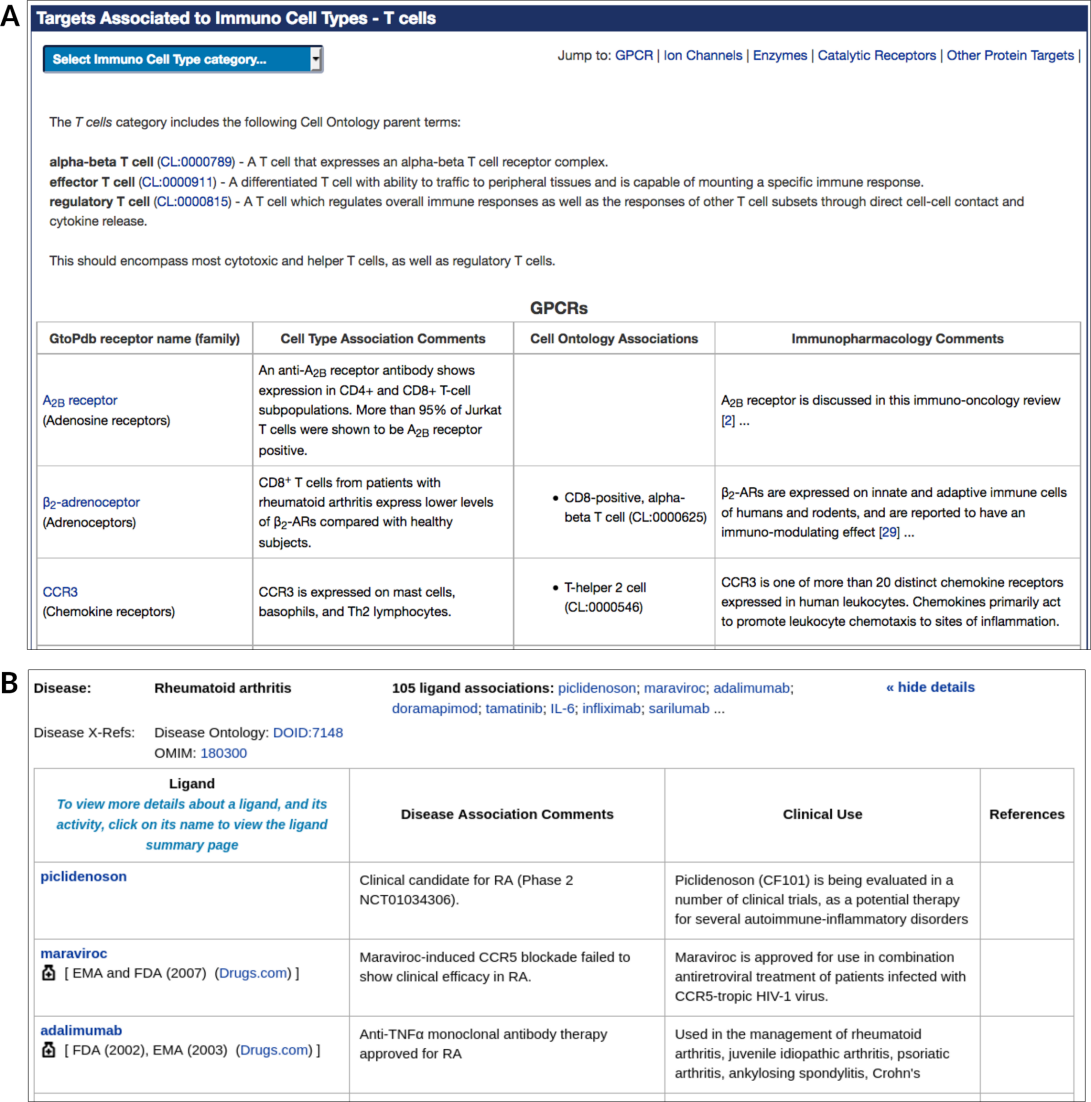
Displaying GtoImmuPdb data. (**A**) Shows the display of GtoImmuPdb cell type to target associations, here showing T cells. This layout is also used for the display of process association data. (**B**) Shows the display of ligand disease associations, showing the first three ligands for rheumatoid arthritis.

Users can click on the portal's cell types panel to access a list of targets associated to each cell type (www.guidetoimmunopharmacology.org/GRAC/ObjectCelltypeAssocListForward?immcelltype=2) (Figure 6A). This is very similar to the process list described above. Curators’ comments are displayed next to CO terms and IDs for each target. It includes links to each target class, and a pull-down menu to switch between cell type categories.

### Disease associations

The portal also links to lists of curated immunological disease associations to either targets or ligands. Figure 6B shows the display for ligands associated to immunological diseases. Users can switch between viewing targets or ligands via a tab at the top of the page. Diseases are listed in alphabetical order, with external references to OMIM, Orphanet and the Disease Ontology.

A single disease may be associated with many different ligands, although usually not as many targets. Therefore, the page keeps the full list of targets and ligands associated with each disease hidden on initial view. The total number of targets or ligands associated with the disease is shown, along with a preview of the names of the first few targets or ligands. A toggle link can be clicked to expand the section to list all associations. There are controls on the page to hide or show all the disease associations if required. In the expanded view, target associations show the name of the target, curator comments and lists of approved drugs (ligands which are currently, or have been in the past, approved for human clinical use by a regulatory agency) that interact with the target. This list is restricted to only those drugs for which the target is classed as a primary target of the compound. When viewing disease associations to ligands in the expanded view, it lists the ligand name (linked to the ligand summary page) and curator comments.

### Targets

**Figure 7.**
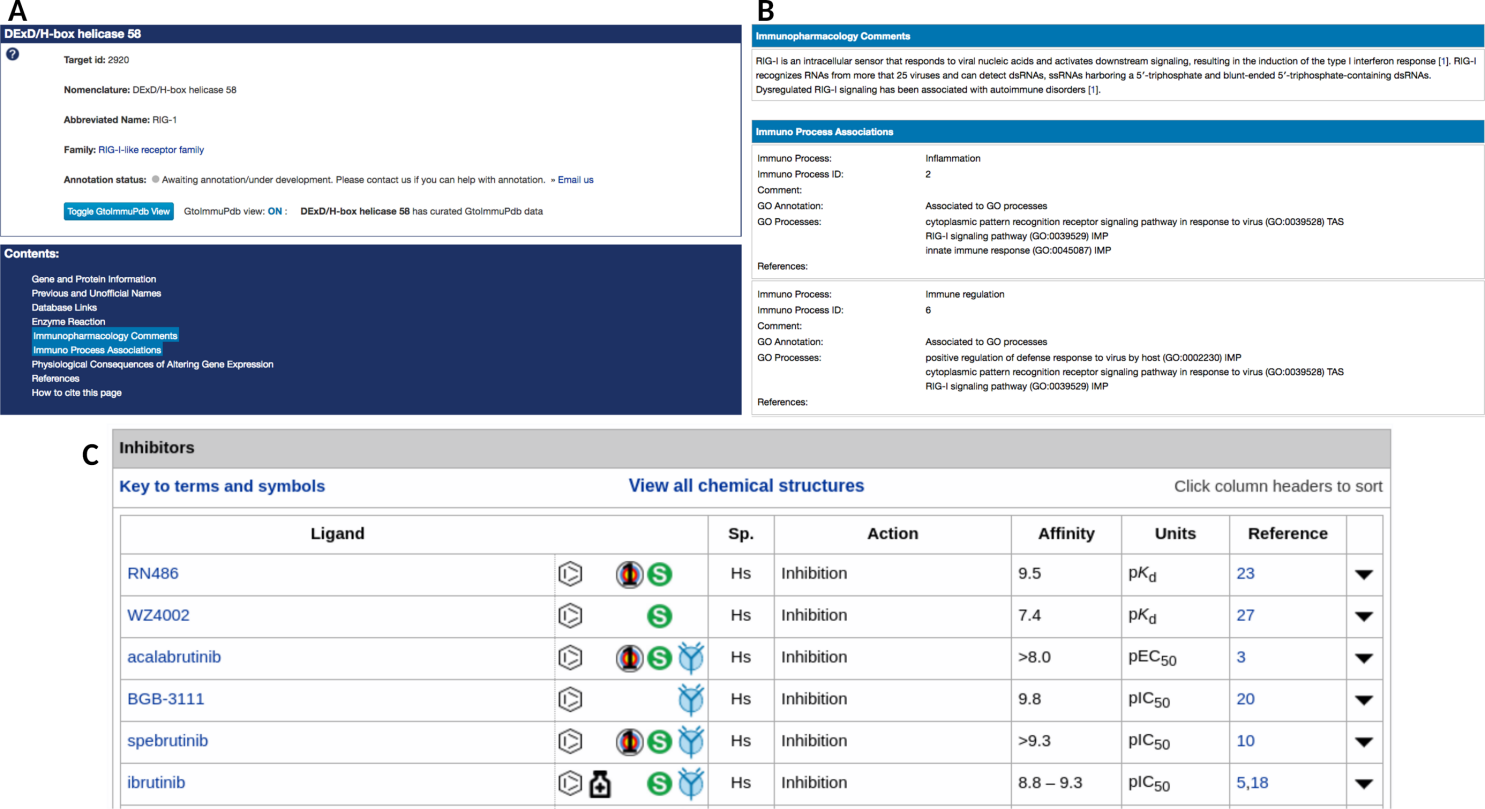
Showing the target detailed view page with immunological data highlighted. (**A**) Top section of the detailed view page for RIG-1 (DExD/H-Box helicase 58) with immunopharmacology content links highlighted. (**B**) Immunopharmacology data sections on the detailed view page. (**C**) Ligand binding data (from Bruton tyrosine kinase) showing the new immunological icon in blue to highlight ligands also tagged in GtoImmuPdb.

Targets are browsed in GtoPdb via hierarchical trees of protein families. This display has been enhanced for GtoImmuPdb by highlighting families of immunological relevance in blue (Figure 1B). Likewise, when viewing a family page, any individual targets of immunological relevance are also highlighted. New toggle buttons allow users to switch between GtoPdb and GtoImmuPdb views. The detailed view page for a target has been extended to include the new immunological data types incorporated for GtoImmuPdb. The same data are displayed in both views, but in the GtoImmuPdb view the new sections are highlighted, helping to alert users to the content. These include general comments on immunopharmacology as well as any process, cell type or disease associations (Figure 7 A and B). Additionally, within the existing sections of the detailed view page that list ligand interactions, a new GtoImmuPdb logo is displayed where the ligand has been tagged of relevance to immunopharmacology (Figure 7C).

### Ligands

Modifications have been made to the way ligand categories are displayed under GtoImmuPdb. The ligands panel on the portal prioritizes the immuno ligand and antibody categories (likely to be of greater interest to immunologists). When viewing the ligand list page, a new ‘immuno’ ligands category has been added as a tab and when viewing any category under the GtoImmuPdb view, only ligands tagged (see ‘Content and curation’ section) in GtoImmuPdb are displayed. Again, a toggle allows the user to switch between GtoPdb and GtoImmuPdb views. As on target pages, relevant ligands are highlighted using the new immunopharmacology icon. Individual ligand summary pages also now have a tab that includes immunopharmacology-related data specific to the ligand. At present, this includes curator comments, disease associations and references.

### Searching GtoImmuPdb

Search mechanisms have been extended to incorporate all additional immunological data, including process, cell type and disease terms, ontological definitions and ontology IDs. We have also modified the search algorithm when searching via the GtoImmuPdb portal to increase the ranking of results likely to be of greater immunological relevance. We applied a weighting to certain database fields so that entities matching on those fields will appear higher up the list of results. The weighting is only applied when searching from a GtoImmuPdb page, not from the standard GtoPdb pages and is based on the amount of immunological data curated against an entity. For example, targets that have process, cell type and disease data annotated against them will rank higher than targets with only process data. This is in addition to existing search weightings—so exact matches (to target or ligand name for example) will still get a higher score.

### Help and tutorials

We encourage users to familiarize themselves with the new data and navigation of the GtoImmuPdb portal by browsing the help pages (www.guidetoimmunopharmacology.org/immuno/immunoHelpPage.jsp) and the specific tutorial (www.guidetoimmunopharmacology.org/immuno/docs/GtoImmuPdb_Tutorial.pdf). The help pages also contain links to download sets of GtoImmuPdb data. We have also added popup help guides on the main portal for each major data type.

## COLLABORATIONS, CONNECTIVITY AND INTEROPERABILITY

### ELIXIR

ELIXIR is the European infrastructure established specifically for the sharing and sustainability of life science data (www.elixir-europe.org/). The UK node, ELIXIR-UK (www.elixir-uk.org/), expanded in 2015/16, sought to identify resources that were representative of the UK bioinformatics community (33) and bring the benefit of participation to a wider range of UK institutions. Falling within ELIXR’s strategic theme of human health and disease, GtoPdb already has links with other ELIXIR resources, including UniProtKB (where we regularly update cross-references), and is in a good position to interoperate with other resources through our use of standard ontologies and identifiers (UniProtKB Accessions, nomenclature (as assigned by NC-IUPHAR committees in collaboration with HGNC), Ensembl IDs, PubMed IDs and PDB IDs). We see to improve our interoperability and FAIR (Findable, Accessible, Interoperable, Reusable) compliance (34) by producing GtoPdb in Resource Description Framework (RDF) format, thereby making the data and the ontologies used machine readable.

### RDF

**Figure 8.**
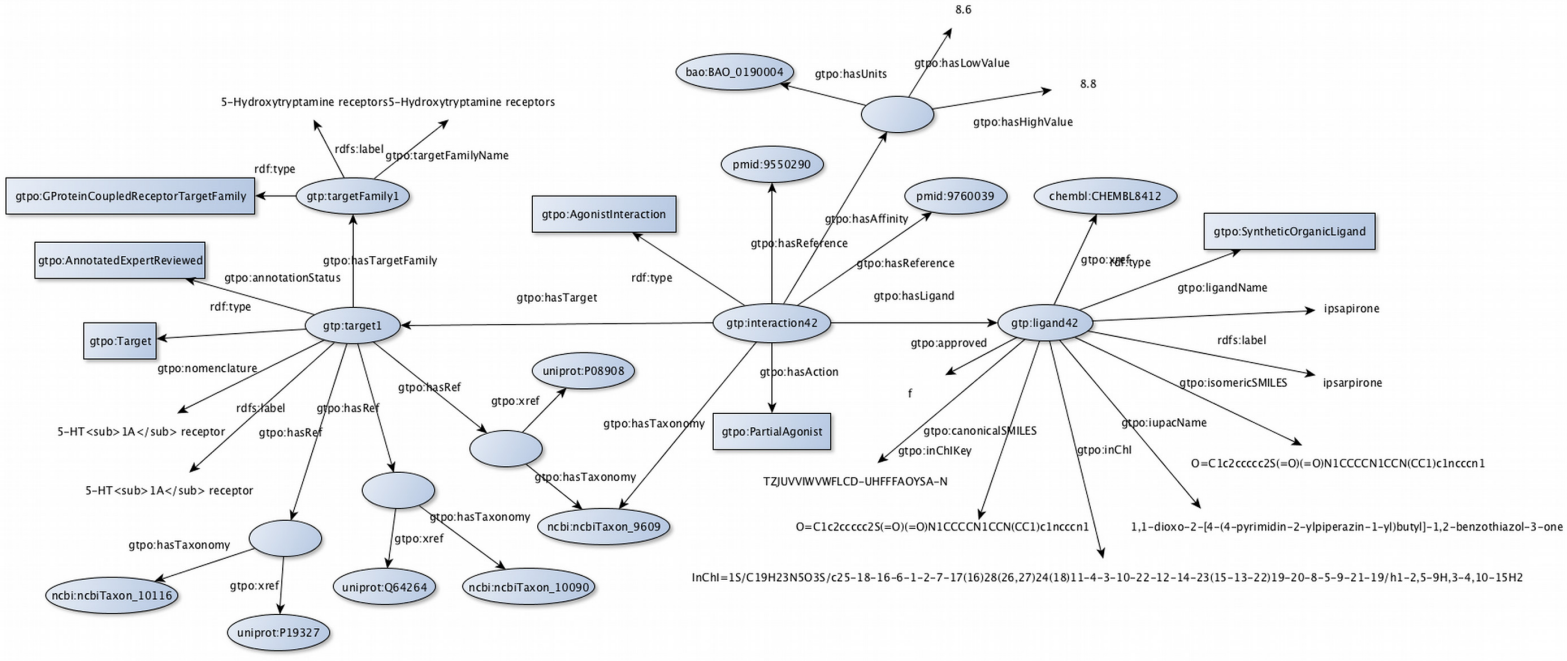
Schematic diagram illustrating the RDF relationship between the target 5-HT_1A_ receptor and the ligand Ipsapirone.

Over the last year, work has been carried out to design and implement an RDF version of a subset of the GtoPdb dataset, now available from (www.guidetopharmacology.org/downloads.jsp). The RDF data generation has been focussed on the main target–ligand interaction data. To ensure FAIR compliance machine-readable metadata have been generated in accordance with the W3C Health Care and Life Sciences Community (HCLS) Profile (35). As an example of RDF modelling, the interaction between the target 5-HT_1A_ receptor and the ligand Ipsapirone is shown in Figure 8. In addition to the already stated aim of making GtoPdb FAIR compliant, it was also planned as a way to include the GtoPdb data into the Open PHACTS Drug Discovery Platform (36,37).

We have developed an ontology (http://rdf.guidetopharmacology.org/ns/gtpo), that represents the schema of the RDF data, in accordance with current best practice (38) using the ‘Indirect reuse of ontology design patterns and alignments’ ontology design pattern (39). The units used for the affinity values in the interactions are reused from the BioAssay Ontology (BAO) (40). Working with the maintainers of BAO new entries have been added for pK_B_, pK_i_ and pK_d_. Defining our own core ontology allowed us to: (i) ensure that the terms capture the actual interpretation in GtoPdb – the terms were modelled around the existing GtoPdb glossary (http://www.guidetopharmacology.org/helpPage.jsp#glossary); (ii) eliminate potentially unstable external dependencies. The downside of the approach is that the terms are not immediately interoperable, for example with the ChEMBL terms, except via the use of the equivalence mappings that are published alongside the ontology. The RDF data are generated from the corresponding released version of the GtoPdb database. This ensures that the RDF data are consistent with the database content and do not require the maintenance of a second database. Three relational-to-RDF (R2RM, (41)) mapping files have been created that control the generation of the RDF data—one each for targets, ligands and interactions respectively. These are available from https://github.com/HW-SWeL/GTP-RDF/tree/master/Morph/Guide2Pharma/R2RML. We execute the mappings using the Morph RDB framework (42). Again, following best practice (43), a table identifier scheme has been established for the RDF data. Another key aspect of the generation process is the creation of machine-readable metadata. The metadata capture the provenance information between the RDF version of the data and the relational database version that was used to create it. The metadata comply with the W3C HCLS Community Profile to the SHOULD level and this has been verified with the Validata tool (http://hw-swel.github.io/Validata).

We are in the process of providing linked data pages for each resource in the RDF data, i.e. the URL used to identify each target, ligand and interaction in the RDF will become dereferenceable. Additionally, we will be providing a SPARQL endpoint with a set of example queries to help exploit the RDF data and enable deeper analysis of the GtoPdb data in conjunction with other linked data datasets. In the future, we will provide link-sets that capture the database cross-references contained in the GtoPdb as well as extend coverage of the data.

### Database links

The current set of database link-outs from GtoPdb target and ligand pages can be viewed at www.guidetopharmacology.org/helpPage.jsp#databaseLinks. New links since 2016 on the ligand side include UniChem, which cross-references between chemical structure identifiers from different databases (44). On the target side, there are links to the SLC Transporter Database (http://slc.bioparadigms.org/) with information on transporter functional genomics, CATH structural domains and superfamilies (45) and the Human Protein Atlas tissue expression database (46), these latter two being ELIXIR core resources (47). We have also ensured cross-links exist between GtoPdb and the Pharmacology Education Website (PEP, www.pharmacologyeducation.org/) and SynPharm (http://synpharm.guidetopharmacology.org/), the database of ligand-binding sequences.

We ensure that the cross-links are regularly refreshed through formal and informal contacts with database providers. Of note are the links to RCSB Protein Data Bank (PDB) (www.rcsb.org) (48) ligands. The acceleration at which pharmacological targets are being co-crystallized with ligands caused us to introduce PDB InChIKey look-ups as part of our database update procedure, to ensure that links are up-to-date in each release. Consequent to the creation of peptide ligand family pages, we also updated our reciprocal HGNC links to include human genes encoding peptide ligands. We encourage in-links from other resources, although it is difficult to discern the extent of these unless we are directly informed or cited. A recent survey (https://blog.guidetopharmacology.org/2016/03/30/collation-and-assessment-of-gtopdb-in-links) found these were over 20 in number and we also not that we have recently been incorporated into an internal-only resource, the CHEMGENIE database from Merck & Co (49).

### Journal to database connectivity

GtoPdb has continued to enhance its connectivity to journals. Much of our endeavours since our last update (5) have been described in our blog (https://blog.guidetopharmacology.org/2017/10/18/gtopdb-nar-database-issue-2018-journal-to-database-connectivity-and-journal-to-gtopdb-links/). This includes details on PubMed ID statistics in curated GtoPdb content. The same post also shows how engagement with *British Journal of Pharmacology* (BJP) has extended the previous provision of live out-links (50), to now include in line links, rather than the previous method of adding separate tables to manuscripts.

### External profile

We use a number of strategies to disseminate information about GtoPdb. Our NC-IUPHAR newsletter is prepared in sync with database releases and sent to subscribers and committee members with articles about the database and hot topics in pharmacology. Social media (Twitter, Facebook and LinkedIn) is one of our primary methods of outreach to announce database updates, publications of interest, upcoming events and IUPHAR reviews. Our blog (https://blog.guidetopharmacology.org/) is used to provide detailed descriptions of database releases, technical updates and commentary on hot topics in pharmacology. For example, the development of the Guide to IMUNOPHARMACOLOGY is described in a series of technical blog posts (https://blog.guidetopharmacology.org/category/guide-to-immunopharmacology/). We also share posters and presentations made by the team on our SlideShare account (www.slideshare.net/GuidetoPHARM). We track user interactions via Google analytics which shows a monthly average of ∼29 500 sessions from 19 500 users, over the 6 months from April 2017 to September 2017. We have also produced our third edition of The Concise Guide to PHARMACOLOGY 2017/18 that includes links to 1679 targets and 3524 ligands (www.guidetopharmacology/concise).

## FUTURE DIRECTIONS

Guide to IMMUNOPHARMACOLOGY is in its beta phase: we expect the portal and website to be completed by Spring 2018 when the first full public release will be available. At present, most features of the interface are in place. We have undertaken a short phase of user-testing; an exercise we plan to repeat before the full public release. This will identify any aspects of the design and navigation that need improvement, and features that users would like to see implemented. In response to previous user feedback we will also consider a graphical-based navigation system to improve user-friendliness and enhance the look of the website.

New data curation will continue until Autumn 2018, with a focus on target associations to disease and cell types that are currently under-represented in the database. We will also continue to assign further targets to top-level immunological process categories by looking at their GO annotations and supporting evidence.

We will continue the development of our RDF platform with short-term priorities of establishing a linked data explorer and a SPARQL end-point to enable queries across the data. Making the RDF publicly available allows integration into other semantic databases (both proprietary and non-proprietary).

We are also working with Bioschemas (http://bioschemas.org/) to add schema.org semantic mark-up to GtoPdb, which would make it easier for search engines to find, collate and analyse the data (51).

Many of the main use-cases and features in GtoPdb will be elaborated on in a forthcoming paper in Current Protocols in Bioinformatics (manuscript in preparation).

A new, sister resource to the main GtoPdb called SynPharm (http://synpharm.guidetopharmacology.org/) has been developed as a database of drug-responsive protein sequences, derived from the interactions in GtoPdb and using data from the RCSB PDB. SynPharm is an open-access, web-based tool that integrates pharmacological and ligand–protein binding information to present data on the drug-binding domains of proteins in a manner useful to synthetic biologists. It will be kept up-to-date in line with GtoPdb and we are preparing a paper detailing the resource.

## DATA ACCESS

GtoPdb and GtoImmuPdb are available online at http://www.guidetopharmacology.org and http://www.guidetoimmunopharmacology.org, respectively. Both are licensed under the Open Data Commons Open Database License (ODbL) (http://opendatacommons.org/licenses/odbl/), and the contents are licensed under the Creative Commons Attribution-ShareAlike 3.0 Unported license (http://creativecommons.org/licenses/by-sa/3.0/). Recommendations for accessing and downloading data, and linking to us remain as reported in 2016. We provide various options for download, and users are welcome to contact us (enquiries@guidetopharmacology.org) for additional datasets and file formats. We provide a dump file of the full PostgreSQL database (http://www.postgresql.org/) but we no longer provide a MySQL version as standard (although we would be glad to provide this on request). We also provide RDF flat files as described above (available from www.guidetopharmacology.org/downloads.jsp), which users can load into a local triple store and perform SPARQL queries across the data. Our REST web services are available at http://www.guidetopharmacology.org/webServices.jsp and provide computational access to data in JavaScript Object Notation (JSON) format. In response to user feedback we recently expanded and revised our web services and made speed improvements. We added services to allow direct querying of interaction data and references. For example, users can now obtain a complete set of target–ligand interactions with many options to filter this list, including by target or ligand type, binding affinity, drug approval status or chemical structure. We encourage users to get in touch if they download data in any format, both for further advice and so we are aware of applications using GtoPdb data.

## CITING THE RESOURCE

Please cite this article rather than the previous ones; citation advice for specific target pages appears on the website. Please refer to our resources on first mention by full correct name (IUPHAR/BPS Guide to PHARMACOLOGY and IUPHAR Guide to IMMUNOPHARMACOLOGY) including the capitalization. For subsequent abbreviation please use GtoPdb and GtoImmuPdb and specify the release version number (this can be found on our About page (http://www.guidetopharmacology.org/about.jsp#content)).

## References

[1] Harmar A.J., Hills R.A., Rosser E.M., Jones M., Buneman O.P., Dunbar D.R., Greenhill S.D., Hale V.A., Sharman J.L., Bonner T.I. IUPHAR-DB: the IUPHAR database of G protein-coupled receptors and ion channels. Nucleic Acids Res. 2009; 37:D680–D685. 18948278 10.1093/nar/gkn728 PMC2686540

[2] Sharman J.L., Benson H.E., Pawson A.J., Lukito V., Mpamhanga C.P., Bombail V., Davenport A.P., Peters J.A., Spedding M., Harmar A.J. IUPHAR-DB: updated database content and new features. Nucleic Acids Res. 2013; 41:D1083–D1088. 23087376 10.1093/nar/gks960 PMC3531077

[3] Sharman J.L., Mpamhanga C.P., Spedding M., Germain P., Staels B., Dacquet C., Laudet V., Harmar A.J. IUPHAR-DB: new receptors and tools for easy searching and visualization of pharmacological data. Nucleic Acids Res. 2011; 39:D534–D538. 21087994 10.1093/nar/gkq1062 PMC3013670

[4] Pawson A.J., Sharman J.L., Benson H.E., Faccenda E., Alexander S.P., Buneman O.P., Davenport A.P., McGrath J.C., Peters J.A., Southan C. The IUPHAR/BPS Guide to PHARMACOLOGY: an expert-driven knowledgebase of drug targets and their ligands. Nucleic Acids Res. 2014; 42:D1098–D1106. 24234439 10.1093/nar/gkt1143 PMC3965070

[5] Southan C., Sharman J.L., Benson H.E., Faccenda E., Pawson A.J., Alexander S.P., Buneman O.P., Davenport A.P., McGrath J.C., Peters J.A. The IUPHAR/BPS Guide to PHARMACOLOGY in 2016: towards curated quantitative interactions between 1300 protein targets and 6000 ligands. Nucleic Acids Res. 2016; 44:D1054–D1068. 26464438 10.1093/nar/gkv1037 PMC4702778

[6] Ishii M. Immunology proves a great success for treating systemic autoimmune diseases—a perspective on immunopharmacology: IUPHAR Review 23. Br. J. Pharmacol. 2017; 174:1875–1880. 28299772 10.1111/bph.13784 PMC5466525

[7] Landolina N., Levi-Schaffer F. Monoclonal antibodies: the new magic bullets for allergy: IUPHAR Review 17. Br. J. Pharmacol. 2016; 173:793–803. 26620589 10.1111/bph.13396 PMC4761089

[8] Tiligada E., Ishii M., Riccardi C., Spedding M., Simon H.U., Teixeira M.M., Cuervo M.L., Holgate S.T., Levi-Schaffer F. The expanding role of immunopharmacology: IUPHAR Review 16. Br. J. Pharmacol. 2015; 172:4217–4227. 26173913 10.1111/bph.13219 PMC4556463

[9] Carvalho S., Levi-Schaffer F., Sela M., Yarden Y. Immunotherapy of cancer: from monoclonal to oligoclonal cocktails of anti-cancer antibodies: IUPHAR Review 18. Br. J. Pharmacol. 2016; 173:1407–1424. 26833433 10.1111/bph.13450 PMC4831314

[10] Licastro F., Candore G., Lio D., Porcellini E., Colonna-Romano G., Franceschi C., Caruso C. Innate immunity and inflammation in ageing: a key for understanding age-related diseases. Immun. Ageing. 2005; 2:8. 15904534 10.1186/1742-4933-2-8 PMC1166571

[11] McInnes I.B., Schett G. The pathogenesis of rheumatoid arthritis. N. Engl. J. Med. 2011; 365:2205–2219. 22150039 10.1056/NEJMra1004965

[12] O'Shea J.J., Holland S.M., Staudt L.M. JAKs and STATs in immunity, immunodeficiency, and cancer. N. Engl. J. Med. 2013; 368:161–170. 23301733 10.1056/NEJMra1202117 PMC7604876

[13] Perry V.H., Newman T.A., Cunningham C. The impact of systemic infection on the progression of neurodegenerative disease. Nat. Rev. Neurosci. 2003; 4:103–112. 12563281 10.1038/nrn1032

[14] Di Marco Barros R., Roberts N.A., Dart R.J., Vantourout P., Jandke A., Nussbaumer O., Deban L., Cipolat S., Hart R., Iannitto M.L. Epithelia use butyrophilin-like molecules to shape organ-specific gammadelta T cell compartments. Cell. 2016; 167:203–218. 27641500 10.1016/j.cell.2016.08.030 PMC5037318

[15] Gray K.A., Seal R.L., Tweedie S., Wright M.W., Bruford E.A. A review of the new HGNC gene family resource. Hum. Genomics. 2016; 10:6. 26842383 10.1186/s40246-016-0062-6 PMC4739092

[16] Yates B., Braschi B., Gray K.A., Seal R.L., Tweedie S., Bruford E.A. Genenames.org: the HGNC and VGNC resources in 2017. Nucleic Acids Res. 2017; 45:D619–D625. 27799471 10.1093/nar/gkw1033 PMC5210531

[17] Southan C., Sitzmann M., Muresan S. Comparing the chemical structure and protein content of ChEMBL, DrugBank, Human Metabolome Database and the Therapeutic Target Database. Mol. Inform. 2013; 32:881–897. 24533037 10.1002/minf.201300103 PMC3916886

[18] Law V., Knox C., Djoumbou Y., Jewison T., Guo A.C., Liu Y., Maciejewski A., Arndt D., Wilson M., Neveu V. DrugBank 4.0: shedding new light on drug metabolism. Nucleic Acids Res. 2014; 42:D1091–D1097. 24203711 10.1093/nar/gkt1068 PMC3965102

[19] Ursu O., Holmes J., Knockel J., Bologa C.G., Yang J.J., Mathias S.L., Nelson S.J., Oprea T.I. DrugCentral: online drug compendium. Nucleic Acids Res. 2017; 45:D932–D939. 27789690 10.1093/nar/gkw993 PMC5210665

[20] Altschul S.F., Gish W., Miller W., Myers E.W., Lipman D.J. Basic local alignment search tool. J. Mol. Biol. 1990; 215:403–410. 2231712 10.1016/S0022-2836(05)80360-2

[21] Bento A.P., Gaulton A., Hersey A., Bellis L.J., Chambers J., Davies M., Kruger F.A., Light Y., Mak L., McGlinchey S. The ChEMBL bioactivity database: an update. Nucleic Acids Res. 2014; 42:D1083–D1090. 24214965 10.1093/nar/gkt1031 PMC3965067

[22] Neubig R.R., Spedding M., Kenakin T., Christopoulos A. International union of pharmacology committee on receptor nomenclature and drug classification. XXXVIII. Update on terms and symbols in quantitative pharmacology. Pharmacol. Rev. 2003; 55:597–606. 14657418 10.1124/pr.55.4.4

[23] Good B.M., Tennis J.T., Wilkinson M.D. Social tagging in the life sciences: characterizing a new metadata resource for bioinformatics. BMC Bioinformatics. 2009; 10:313. 19781082 10.1186/1471-2105-10-313 PMC2760536

[24] Ashburner M., Ball C.A., Blake J.A., Botstein D., Butler H., Cherry J.M., Davis A.P., Dolinski K., Dwight S.S., Eppig J.T. Gene ontology: tool for the unification of biology. The Gene Ontology Consortium. Nat. Genet. 2000; 25:25–29. 10802651 10.1038/75556 PMC3037419

[25] The Gene Ontology Consortium Expansion of the Gene Ontology knowledgebase and resources. Nucleic Acids Res. 2017; 45:D331–D338. 27899567 10.1093/nar/gkw1108 PMC5210579

[26] Bard J., Rhee S.Y., Ashburner M. An ontology for cell types. Genome Biol. 2005; 6:R21. 15693950 10.1186/gb-2005-6-2-r21 PMC551541

[27] Lovering R.C., Camon E.B., Blake J.A., Diehl A.D. Access to immunology through the Gene Ontology. Immunology. 2008; 125:154–160. 18798919 10.1111/j.1365-2567.2008.02940.x PMC2561138

[28] The UniProt Consortium UniProt: the universal protein knowledgebase. Nucleic Acids Res. 2017; 45:D158–D169. 27899622 10.1093/nar/gkw1099 PMC5210571

[29] Gangwar R.S., Landolina N., Arpinati L., Levi-Schaffer F. Mast cell and eosinophil surface receptors as targets for anti-allergic therapy. Pharmacol. Therapeut. 2017; 170:37–63. 10.1016/j.pharmthera.2016.10.010 27773785

[30] Artis D., Spits H. The biology of innate lymphoid cells. Nature. 2015; 517:293–301. 25592534 10.1038/nature14189

[31] Amberger J.S., Bocchini C.A., Schiettecatte F., Scott A.F., Hamosh A. OMIM.org: Online Mendelian Inheritance in Man (OMIM(R)), an online catalog of human genes and genetic disorders. Nucleic Acids Res. 2015; 43:D789–D798. 25428349 10.1093/nar/gku1205 PMC4383985

[32] Kibbe W.A., Arze C., Felix V., Mitraka E., Bolton E., Fu G., Mungall C.J., Binder J.X., Malone J., Vasant D. Disease Ontology 2015 update: an expanded and updated database of human diseases for linking biomedical knowledge through disease data. Nucleic Acids Res. 2015; 43:D1071–D1078. 25348409 10.1093/nar/gku1011 PMC4383880

[33] Hancock J.M., Game A., Ponting C.P., Goble C.A. An open and transparent process to select ELIXIR Node Services as implemented by ELIXIR-UK [version 2; referees: 2 approved, 1 approved with reservations]. F1000Research. 2017; 5(ELIXIR):2894 doi:10.12688/f1000research.10473.2. 10.12688/f1000research.10473.1 PMC5265702 28149502

[34] Wilkinson M.D., Dumontier M., Aalbersberg I.J., Appleton G., Axton M., Baak A., Blomberg N., Boiten J.W., da Silva Santos L.B., Bourne P.E. The FAIR Guiding Principles for scientific data management and stewardship. Sci. Data. 2016; 3:160018. 26978244 10.1038/sdata.2016.18 PMC4792175

[35] Dumontier M., Gray A.J.G., Marshall M.S., Alexiev V., Ansell P., Bader G., Baran J., Bolleman J.T., Callahan A., Cruz-Toledo J. The health care and life sciences community profile for dataset descriptions. PeerJ. 2016; 4:e2331. 27602295 10.7717/peerj.2331 PMC4991880

[36] Williams A.J., Harland L., Groth P., Pettifer S., Chichester C., Willighagen E.L., Evelo C.T., Blomberg N., Ecker G., Goble C. Open PHACTS: semantic interoperability for drug discovery. Drug Discov. Today. 2012; 17:1188–1198. 22683805 10.1016/j.drudis.2012.05.016

[37] Gray A.J.G., Groth P., Loizou A., Askjaer S., Brenninkmeijer C.Y.A., Burger K., Chichester C., Evelo C.T., Goble C.A., Harland L. Applying linked data approaches to pharmacology: architectural decisions and implementation. Semantic Web. 2014; 5:101–113.

[38] Courtot M., Malone J., Mungall C.J. Ten simple rules for biomedical ontology development. CEUR Workshop Proceedings. 2016; 1747:IT404.

[39] Presutti V., Lodi G., Nuzzolese A., Gangemi A., Peroni S., Asprino L. Comyn-Wattiau I, Tanaka K, Song IY, Yamamoto S, Saeki M The Role of Ontology Design Patterns in Linked Data Projects. Conceptual Modeling. ER 2016. Lecture Notes in Computer Science. 2016; 9974:Cham, Switzerland: Springer 113–121.

[40] Abeyruwan S., Vempati U.D., Kucuk-McGinty H., Visser U., Koleti A., Mir A., Sakurai K., Chung C., Bittker J.A., Clemons P.A. Evolving BioAssay Ontology (BAO): modularization, integration and applications. J. Biomed. Semantics. 2014; 5:S5. 25093074 10.1186/2041-1480-5-S1-S5 PMC4108877

[41] Das S., Sundara S., Cyganiak R. R2RML: RDB to RDF Mapping Language. W3C Recommendation. 2012; http://www.w3.org/TR/2012/REC-r2rml-20120927/.

[42] Priyatna F., Corcho O., Sequeda J. Proceedings of the 23rd International Conference on World Wde Web - WWW ’14. 2014; NY: ACM Press 479–490.

[43] McMurry J.A., Juty N., Blomberg N., Burdett T., Conlin T., Conte N., Courtot M., Deck J., Dumontier M., Fellows D.K. Identifiers for the 21st century: How to design, provision, and reuse persistent identifiers to maximize utility and impact of life science data. PLoS Biol. 2017; 15:e2001414. 28662064 10.1371/journal.pbio.2001414 PMC5490878

[44] Chambers J., Davies M., Gaulton A., Papadatos G., Hersey A., Overington J.P. UniChem: extension of InChI-based compound mapping to salt, connectivity and stereochemistry layers. J. Cheminform. 2014; 6:43. 25221628 10.1186/s13321-014-0043-5 PMC4158273

[45] Sillitoe I., Lewis T.E., Cuff A., Das S., Ashford P., Dawson N.L., Furnham N., Laskowski R.A., Lee D., Lees J.G. CATH: comprehensive structural and functional annotations for genome sequences. Nucleic Acids Res. 2015; 43:D376–D381. 25348408 10.1093/nar/gku947 PMC4384018

[46] Uhlen M., Fagerberg L., Hallstrom B.M., Lindskog C., Oksvold P., Mardinoglu A., Sivertsson A., Kampf C., Sjostedt E., Asplund A. Proteomics. Tissue-based map of the human proteome. Science. 2015; 347:1260419. 25613900 10.1126/science.1260419

[47] Durinx C., McEntyre J., Appel R., Apweiler R., Barlow M., Blomberg N., Cook C., Gasteiger E., Kim J.-H., Lopez R. Identifying ELIXIR Core Data Resources [version 2; referees: 2 approved]. F1000Research. 2017; 5(ELIXIR):2422 doi:10.12688/f1000research.9656.2. 10.12688/f1000research.9656.1 PMC5070591 27803796

[48] Rose P.W., Prlic A., Altunkaya A., Bi C., Bradley A.R., Christie C.H., Costanzo L.D., Duarte J.M., Dutta S., Feng Z. The RCSB protein data bank: integrative view of protein, gene and 3D structural information. Nucleic Acids Res. 2017; 45:D271–D281. 27794042 10.1093/nar/gkw1000 PMC5210513

[49] Kutchukian P.S., Chang C., Fox S.J., Cook E., Barnard R., Tellers D., Wang H., Pertusi D., Glick M., Sheridan R.P. CHEMGENIE: integration of chemogenomics data for applications in chemical biology. Drug Discov. Today. 2017; doi:10.1016/j.drudis.2017.09.004. 10.1016/j.drudis.2017.09.004 28917822

[50] McGrath J.C., Pawson A.J., Sharman J.L., Alexander S.P. BJP is linking its articles to the IUPHAR/BPS Guide to PHARMACOLOGY. Br. J. Pharmacol. 2015; 172:2929–2932. 25965085 10.1111/bph.13112 PMC4459013

[51] Gray A.J., Goble C., Jimenez R.C. The Bioschemas Community Bioschemas: From Potato Salad to Protein Annotation. ISWC 2017 Poster Proceedings. 2017; Vienna https://iswc2017.semanticweb.org/paper-579/.

